# Supplementary material for: The m6A reader PRRC2A is essential for meiosis I completion during spermatogenesis
Source: Nat Commun. 2023 Mar 24;14:1636. doi: 10.1038/s41467-023-37252-y (PMC10039029; doi:10.1038/s41467-023-37252-y)
Supplement: Supplementary file 1 — Supplementary Information [file 41467_2023_37252_MOESM1_ESM.pdf]

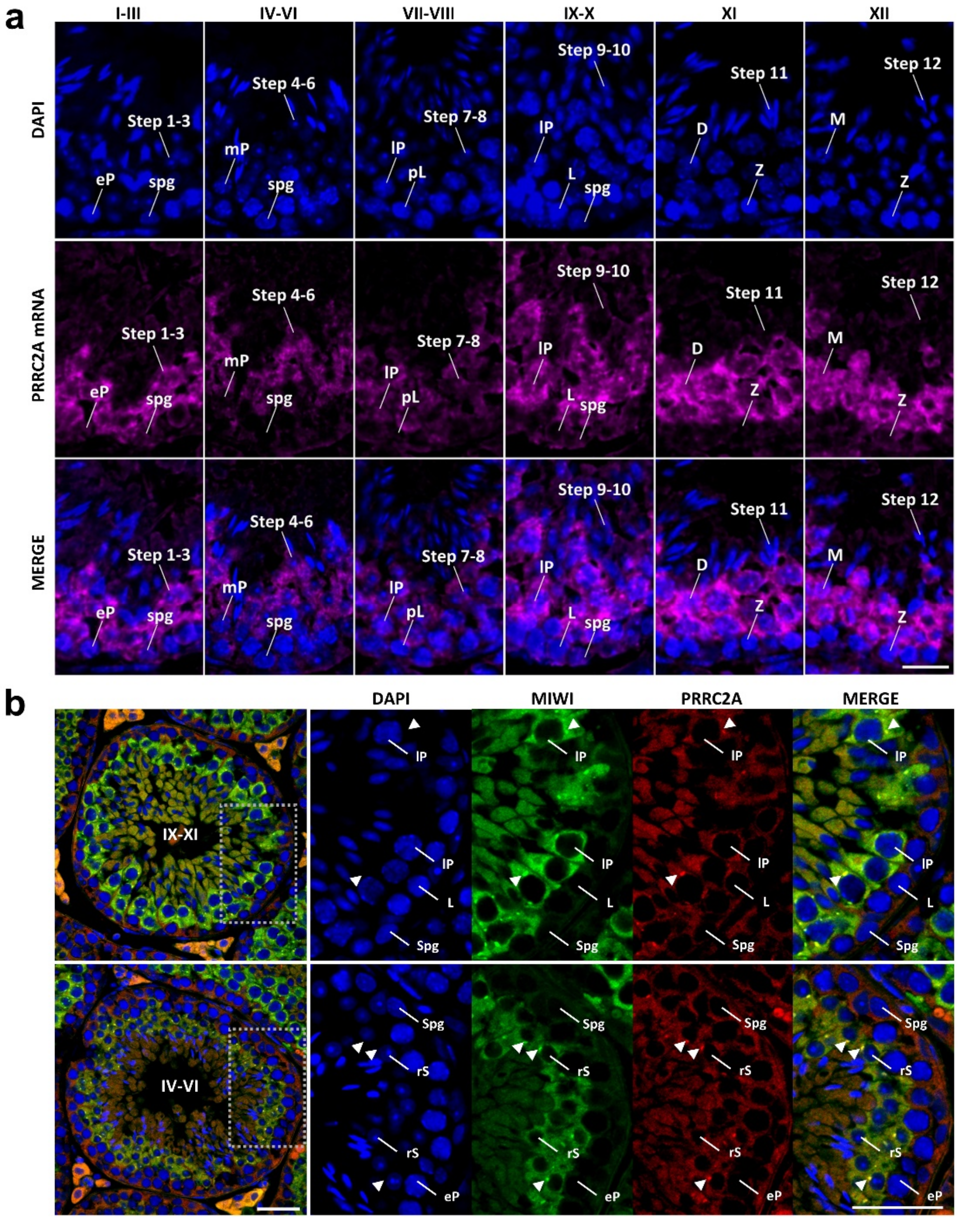

Supplementary Figure 1. Expression pattern of PRRC2A and generation of transgenic mice (a) In situ hybridization of PRRC2A mRNA in P60 wild-type testis sections. Scale bar, 20 μm. (b) Immunostaining of PRRC2A and MIWI in P60 wild-type testis sections. The right panels show enlarged images of indicated areas. Arrowheads indicate chromatoid bodies within indicated cell types. Spg, spermatogonia; pL, preleptotene spermatocyte; L, leptotene spermatocyte; Z, zygotene spermatocyte; eP, early-pachytene spermatocyte; mp, mid-pachytene spermatocyte; lP,

late-pachytene spermatocyte; D, diplotene spermatocytes; rS, round spermatid; Step 1-3, 4-6, 7-8, 8-9, 11, 12 indicate spermatid at different steps. Scale bar, 20 μm.

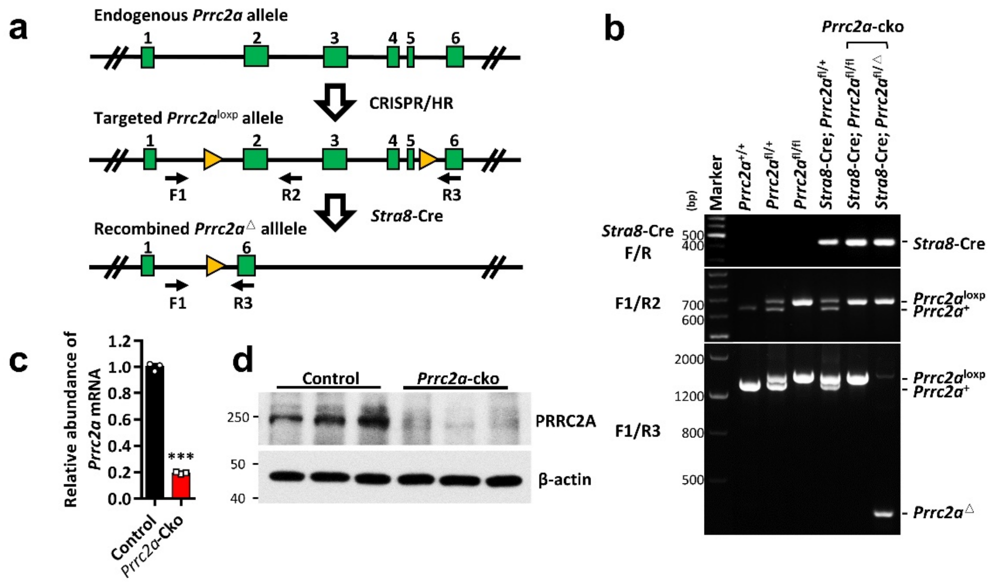

e

	♂/♀	Pregnant female mice	Litters/ abortion	Pups	Mean litter size
Control	5/10	10	8/2	65(40♂25♀)	8.25
Prrc2a-cko	6/12	0	0/0	0	0

Supplementary Figure 2. Generation of transgenic mice and infertility of *Prrc2a*-cko mice (a) Targeting strategy of *Prrc2a*-cko mice. F1, R2, and R3 indicate primers for genotyping (Supplement file 1). (b) Genotyping of *Prrc2a*-cko mice by primers for Stra8-Cre and indicated primers in Figure S2B. (c) qPCR analysis of *Prrc2a* mRNA level in P60 control and *Prrc2a*-cko testes (n = 3). Two-sided student's *t*-test. Error bars, mean ± SEM. ***p < 0.0001. (d) WB analysis of PRRC2A protein level in P60 control and *Prrc2a*-cko testes. (e) Mating and fertility status of control and *Prrc2a*-cko mice.

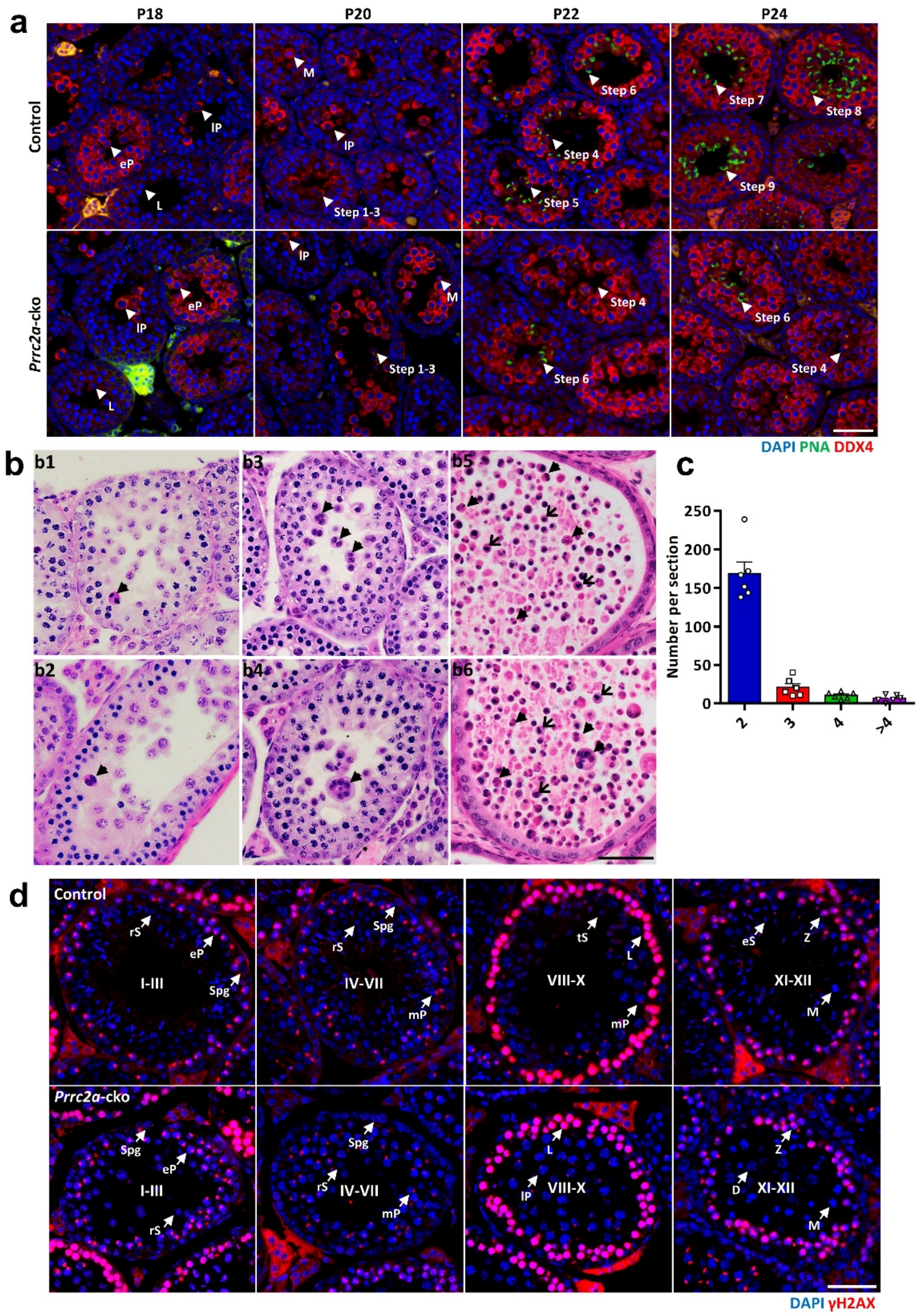

Supplementary Figure 3. PRRC2A deficiency results in developmental arrest and the production of multinucleated cells. (a) Co-staining of DDX4 and PNA in control and *Prrc2a*-cko testis sections of indicated ages. Arrowheads indicate the most advanced type of germ cells in corresponding seminiferous tubules. Scale bar, 50 μm. (b) H&E staining in testes (b1-b4) and epididymis (b5-b6) sections of P60 control and

Prrc2a-cko mice. Arrowheads indicate multinucleated cells. Arrows indicate round spermatids with abnormal nuclear morphology. Scale bar, 50 µm. (c) Numbers of 2-nuclei, 3-nuclei, 4-nuclei and 4-plus-nuclei cells per testis section (n = 6 testes) from P60 *Prrc2a*-cko mice. Error bars, mean ± SEM. (d) Staining of γH2AX in adult control and *Prrc2a*-cko testis sections. Seminiferous tubule stages are indicated. Scale bar, 50 µm. Spg, spermatogonia; L, leptotene spermatocyte; Z, zygotene spermatocyte; eP, early-pachytene spermatocyte; mP, mid-pachytene spermatocyte; lP, late-pachytene spermatocyte; D, diplotene spermatocyte; M, metaphase spermatocyte; rS, round spermatid; tS, transformed spermatid; eS, elongated spermatid; Step 1-3, 4, 5, 6, 7, 8, 9 indicate spermatid at different steps.

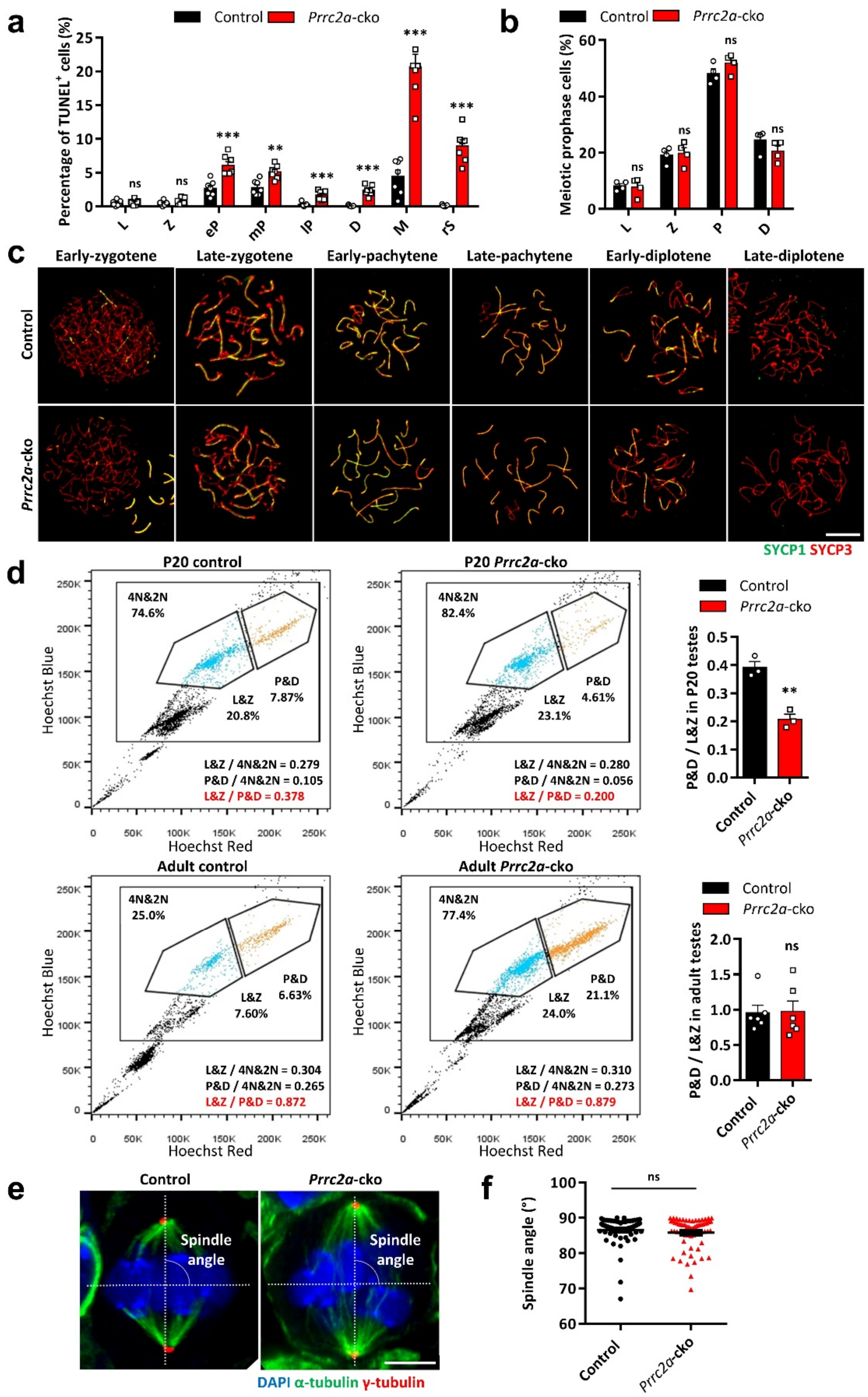

Supplementary Figure 4. Characterization of PRRC2A-null spermatocytes. (a) Percentage of TUNEL$^+$ spermatocytes at indicated stages from control and *Prrc2a*-cko mice (n = 7 biologically independent samples). L, leptotene; Z, zygotene; eP, early-

pachytene; mp, mid-pachytene; lP, late-pachytene; D, diplotene; M, metaphase; rS, round spermatid. Two-sided student's *t*-test. Error bars, mean ± SEM. p = 0.6462 (for L), 0.2396 (for Z), 0.0004 (for eP), 0.0012 (for mP), 0.0003 (for lP), < 0.0001 (for D, M, rS). (b) Proportion of leptotene, zygotene, pachytene, and diplotene spermatocytes in P60 control and *Prrc2a*-cko testes. More than 1000 chromosome spreads of spermatocytes from 4 mice were counted in each group of control and *Prrc2a*-cko. Two-sided student's *t*-test. Error bars, mean ± SEM. p = 0.8316 (for L), 0.8187 (for Z), 0.1736 (for P), 0.1950 (for D). (c) Immunostaining of SYCP1 and SYCP3 on chromosome spreads of control and PRRC2A-null spermatocytes. Scale bar, 10 μm. (d) FACS analysis of testicular cells stained with Hoechst 33,342 and PI from P20 and adult control and *Prrc2a*-cko testes. Squares indicate tetraploid and diploid cells (4N&2N), polygons indicate leptotene & zygotene spermatocytes (L&Z) and pachytene & diplotene spermatocytes (P&D). The percentage of the indicated group in total cells and the ratio between indicated groups are shown. Bar charts show the ratio between L&Z and P&D groups (n = 3 mice for P20, n = 6 for adult). Two-sided student's *t*-test. Error bars, mean ± SEM. ns p = 0.9011, **p = 0.0004. (e, f) Measurement of the average intersection angle between the spindle polarity axis and equatorial plate in control and *Prrc2a*-cko metaphase I spermatocytes (Control, n = 61; *Prrc2a*-cko, n = 64) immunostained with α-tubulin and γ-tubulin. Two-sided student's *t*-test. Error bars, mean ± SEM. ns p = 0.3253. Scale bar, 5 μm.

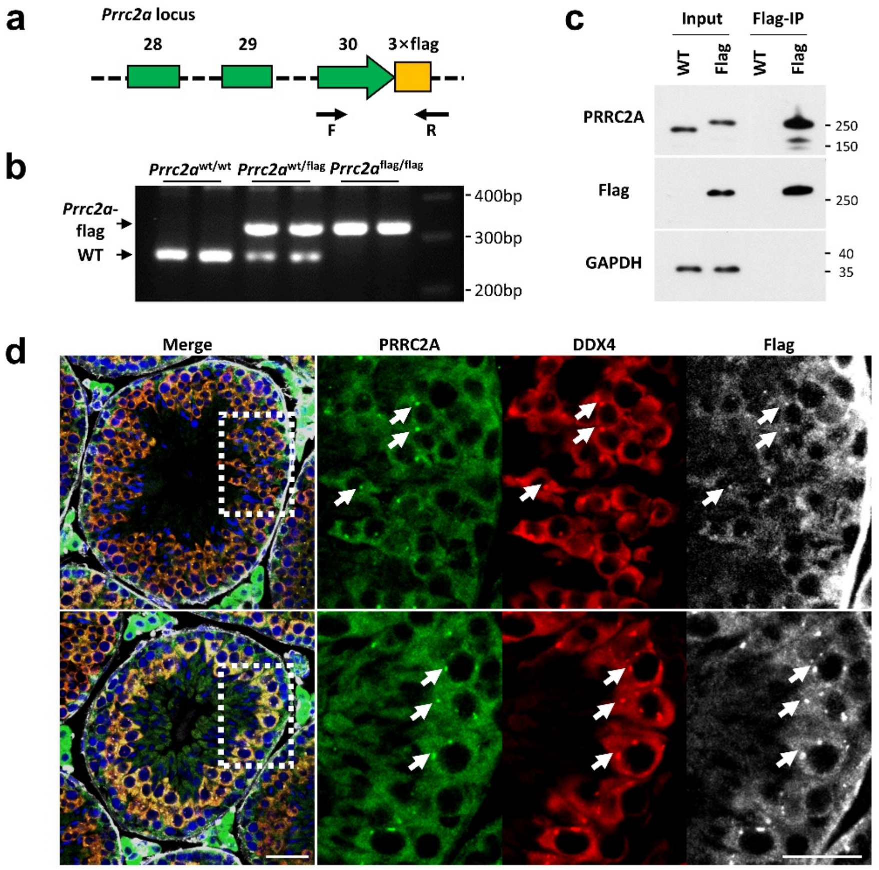

Supplementary Figure 5. Generation of *Prrc2a*-flag transgenic mice and characterizing the expression of PRRC2A-Flag (a) Gene locus of *Prrc2a*-flag mice, 3×flag tag was inserted in the downstream of the last coding exon (exon30). F, R indicate primers for genotyping (Supplementary file 1). (b) Genotyping of *Prrc2a*-flag mice by indicated primers in Figure S5A. (c) IP was performed with testes lysate of adult wild-type (WT) and *Prrc2a*-flag (Flag) mice by anti-Flag antibody, followed by WB detection of indicated proteins. (d) Immunostaining of PRRC2A, DDX4, and Flag in testis sections of P60 *Prrc2a*-flag mice. The right panels show enlarged images of indicated areas. Arrows indicate chromatoid bodies. Scale bar, 20 μm.

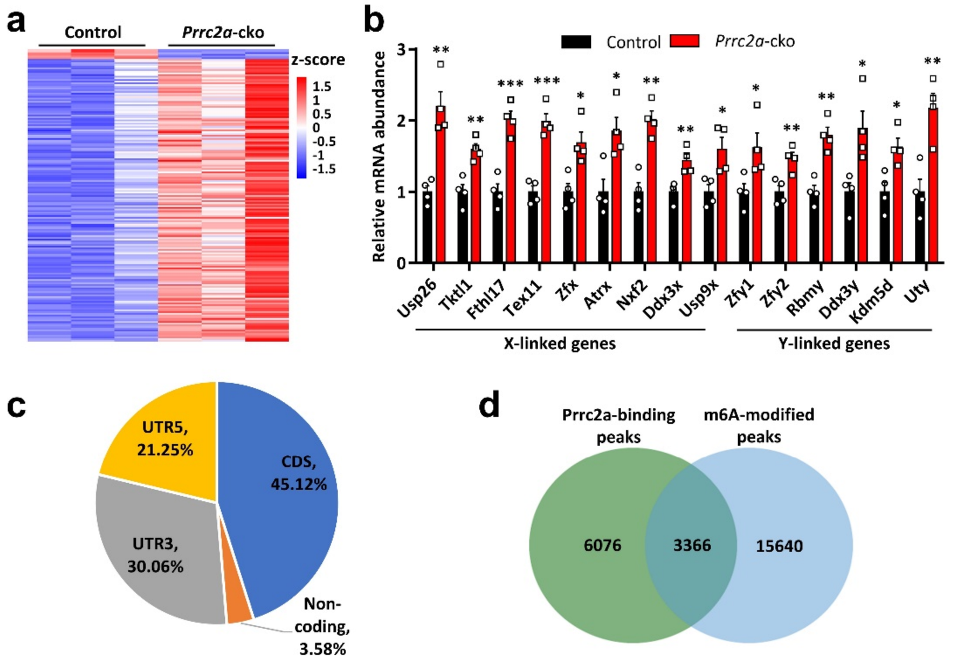

e

Cell type	Up-regulated mRNAs with PRRC2A&m6A	Down-regulated mRNAs with PRRC2A&m6A
Spermatogonia-specific genes	59	2
Spermatocyte-specific genes	0	7
Spermatid-specific genes	0	0
Spermatozoa-specific genes	13	2

Supplementary Figure 6. PRRC2A regulates XY-linked genes and spermatogenic genes (a) Heatmap showing the X (left) and Y (right) -linked genes differentially expressed in PRRC2A-null and control spermatocytes. (b) Representative XY-linked genes are detected by qPCR in PRRC2A-null and control spermatocytes. Two-sided student's *t*-test. Error bars, n = 4 biological repeats, mean ± SEM. p = 0.0004, 0.0122, 0.0171, 0.0012, 0.0088, 0.0219, 0.0412, 0.0085, 0.0016, 0.0187, 0.0150, 0.0052 from left to right. (c) The pie chart shows the distribution region of PRRC2A-binding peaks on transcripts in repeat 2. (d) Overlap of PRRC2A-binding peaks and m6A-modified peaks (refer to Ramesh S. Pillai's paper[11]). (e) Overlap of transcripts with PRRC2A-binding peak containing m6A modification, DTGs in RNA-seq, and cell-type-specific genes.

a

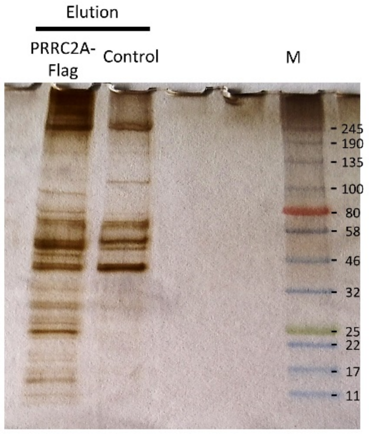

b

Gene name	Mascot score	MW	Matched queries	Matched peptides
Prrc2a	536	38058	8	5
Ybx2	249	69599	8	4
Hspa2	213	105662	4	4
Mthfd1l	199	229063	6	6
Pabpc1	179	70626	4	3
Fxr1	95	174682	1	1
Eif4g3	79	30746	1	1
Csda	76	68648	1	1
Alb	73	76175	2	2
Rpl13	71	24582	1	1
Hspa9	55	73483	1	1
Myl6b	55	22735	1	1
Trf	54	76674	1	1
Cypt12	51	13148	1	1
Hnrnpm	49	77597	1	1
Ybx1	48	35709	1	1
Ldhc	47	35889	1	1
Rims1	45	23995	1	1
Gm4581	45	24143	1	1
Rpl26	44	17139	1	1
Gm5218	44	22499	1	1

c

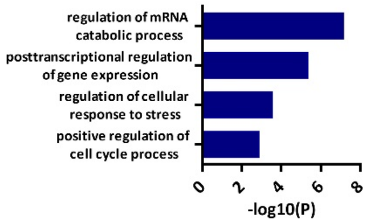

Supplementary Figure 7. Detection of PRRC2A-binding protein by IP-MS (a) IP was performed with testes lysate of *Prrc2a*-flag and wild-type mice by anti-Flag antibody and eluted with Flag peptides. The elution samples were detected by silver staining. (b) The elution samples were analyzed by mass spectrometry. (c) GO terms in biological process categories enriched among PRRC2A-binding proteins identified by IP-MS. The list shows Proteins only detected in the *Prrc2a*-flag group.

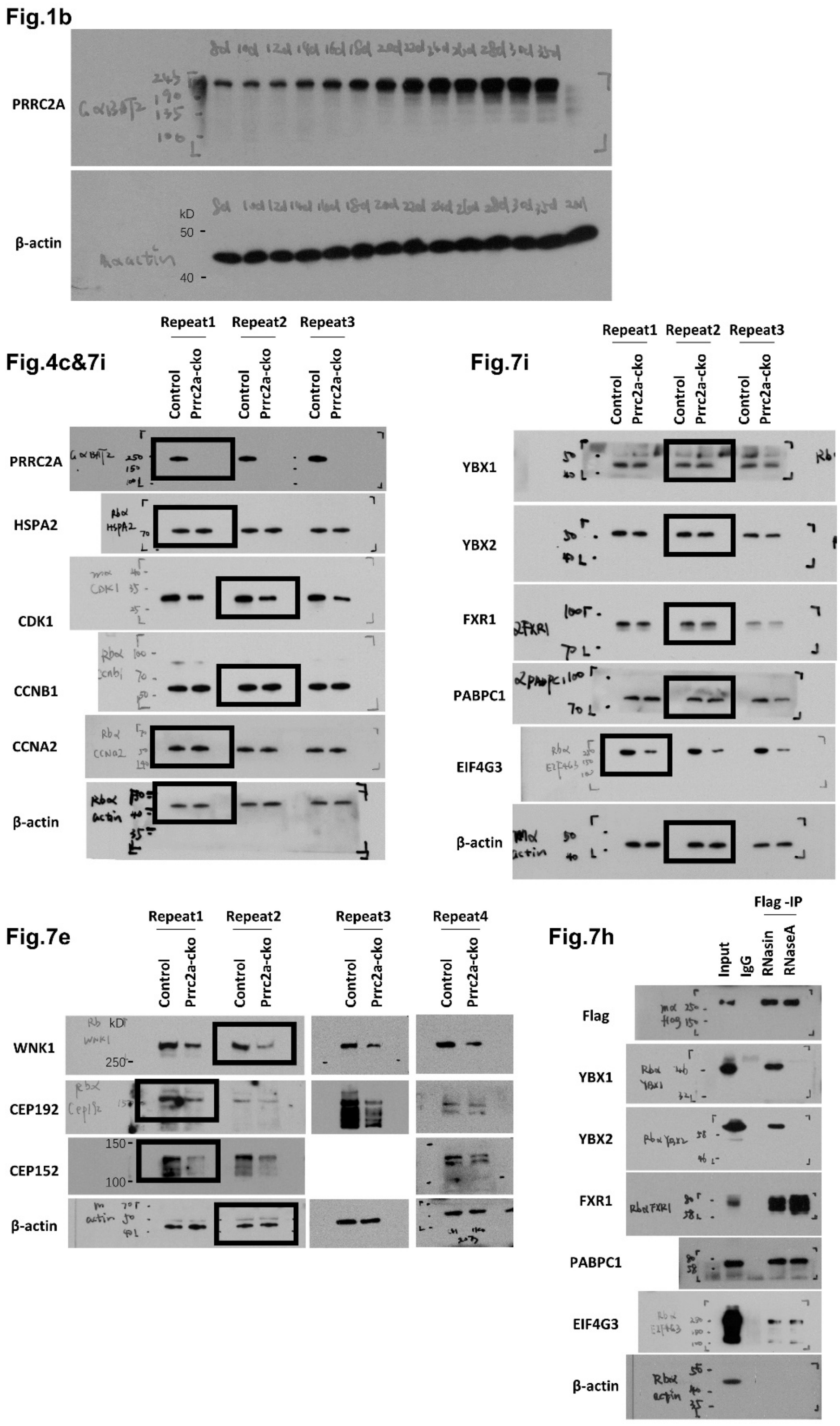

Supplementary Figure 8. Unprocessed images of immunoblotting

PVDF membranes were cut into several small pieces to incubate with different antibodies for immunoblotting. Black boxes indicate images showed in relevant figures.

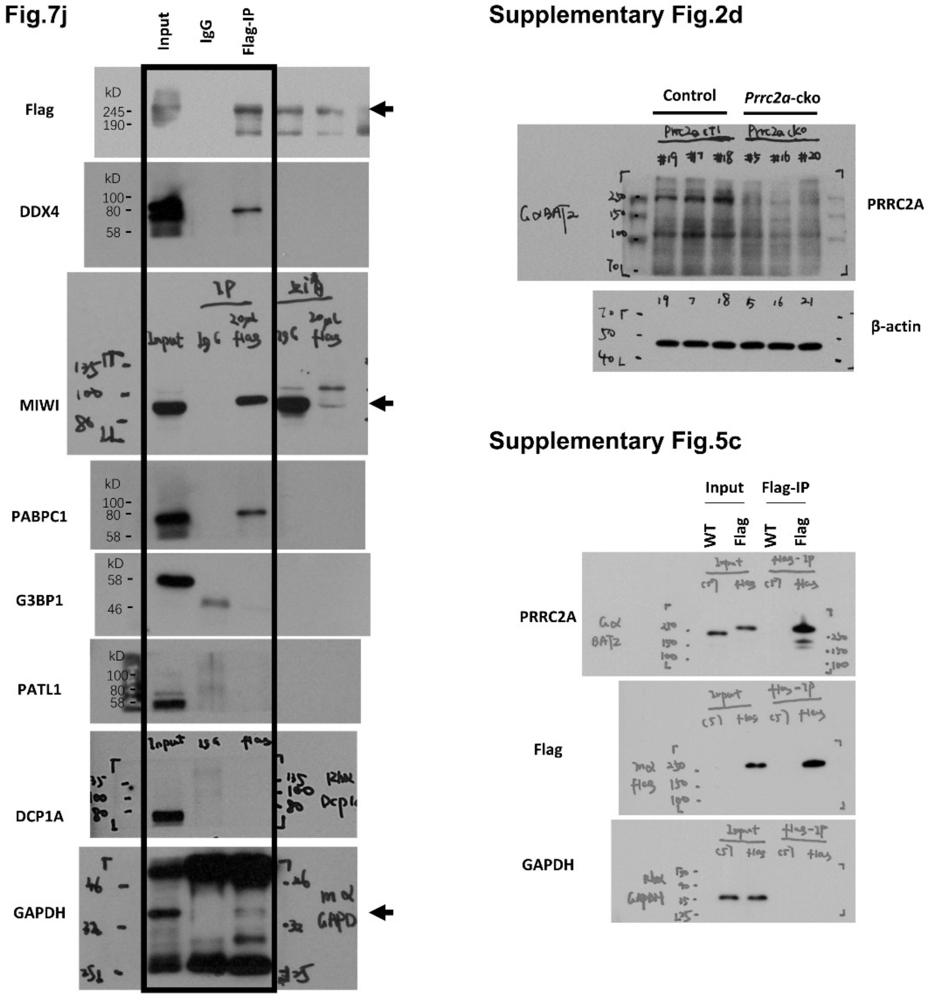

Supplementary Figure 8. Unprocessed images of immunoblotting

PVDF membranes were cut into several small pieces to incubate with different antibodies for immunoblotting. Black boxes indicate images showed in relevant figures.

Supplementary Table 1 List of Primers and gRNAs

**Primers for genotyping**	**Sequence**
Prrc2a-F1	TGGTTACAGGGTGTTGTCGAGGTAGC
Prrc2a-R2	CCATCCACTTGGCTTCCACAGCCCC
Prrc2a-R3	GCGACAGTAGGTTTGATGCC
Stra8-Cre-F	ATGTTTTCACCAATGTCCACGCTCC
Stra8-Cre-R	GGCAGGTTTTGGTGCACAGTCAGC
Prrc2a-flag-F	GAAGGATCGCTGACTTACTCTTCC
Prrc2a-flag-R	GTGAGACTCCAGACCCACAACC
**Primers for qPCR**	
Prrc2a-F	AGGGCAAGTCCTTAGAGATCC
Prrc2a-R	TTCAGGCTTGGAAGGTTGGC
Usp26-F	AATGTAACGAAGGGAGAAGTG
Usp26-R	AGGCTTTGCCTTCTTATCGAG
Ube1x-F	TGACAGAATCCTACAGCTCTAGC
Ube1x-R	CACTGAAGAGTGTGTTCGATGG
Fthl17-F	TCTCGAATGCAGCAGAACTATG
Fthl17-R	GGTCAAAGTAGACTGCCATCG
Tex11-F	GACTGTGGGGTATTGCTTCTG
Tex11-R	CAACTGGCTCCTGTTTTCTGT
Zfx-F	GCAGTGCATGAACAGCAAGTG
Zfx-R	GCCATTCCGGTTTTCAATTCC
Atrx-F	GCTTGTGGACAGCAGGTCAAT
Atrx-R	GTCACGGCTAATATCGTCACTC
Nxf2-F	AAACTGAGGAGGGCGAAACAG
Nxf2-R	AGGGATGAAGGGCTCACTACA
Ddx3x-F	CAGAGTGGAGGAAGTACAGCA
Ddx3x-R	TCACCCCGTGATCCAAAACTG
Usp9x-F	TCCAACAGAATCAGACTTCATCG
Usp9x-R	TGGAAATGCAGGTTCCTCATCT
Zfy1-F	CAGATCAGAGCACTAGCATTCG
Zfy1-R	CTGGCAGTGACATTCTGGTCT
Zfy2-F	ATCCTTTGACAGCCGACATTT
Zfy2-R	CCTCACAGTTGATTCTGGCATC
Rbmy-F	ACCATCCTTTTCAAGAACCAGA
Rbmy-R	TAACTGCAAAGTGTCTCCCAGA
Ddx3y-F	GTTCAGTATTGTAACTGTCTGGCA
Ddx3y-R	TGCTGGCTGATAAACTGAATGT
Kdm5d-F	CCAGGATCTGACGACTTTCTACC
Kdm5d-R	TTCTCCGCAATGGGTCTGATT

Uty-F	ATGCGGATGCTAGCGAAGTT
Uty-R	GAAAGCGGCAGAGGCTATCT
Cep192-F	CCGCATGCTGACATTACTGC
Cep192-R	TCCTTTCCGTCGTAACTGGC
Wnk1-F	GGTGTAAGGTGAGCACAGTGA
Wnk1-R	CACAGCTCACAACCCTCCTC
Dazl-F	TCCTCCTTATCCAAGTTCACCAG
Dazl-R	ACAGTTGTATAAGCCTGGTAGTTA
Gapdh-F	AAGGTCGGTGTGAACGGATTTG
Gapdh-R	TCCTGGAAGATGGTGATGGGCT
RPL6-F	AAGCCCAAGAAGGCGAAGC
RPL6-R	GCAGCCGAGTATTTCCTTTTGTA
**Primers for PRRC2A RIP-qPCR and MeRIP-qPCR**	
Cep192-peak-F	CCATCTGGGAATGCCACCTT
Cep192-peak-R	TACAGCCCAGAAGCCACAAG
Wnk1-peak-F	AGGAGGATAGGAGCCAGCAA
Wnk1-peak-R	AAGGAGCCTCTGCCGATTTC
Dazl-F	TCCTCCTTATCCAAGTTCACCAG
Dazl-R	ACAGTTGTATAAGCCTGGTAGTTA
Gapdh-F	AAGGTCGGTGTGAACGGATTTG
Gapdh-R	TCCTGGAAGATGGTGATGGGCT
**gRNA**	
gRNA for generate *Prrc2a*$^{f/f}$ mice	cagctccctaatcacgcccc
gRNA for generate *Prrc2a*-flag mice	caaggggaactccctcagcg

Supplementary Table 2 List of antibody

**Antibody for immunohistochemistry**			
goat anti-PRRC2A	Santa Cruz	sc-78859	1: 200
rabbit anti-DDX4	Abcam	ab13840	1: 1000
rabbit anti-MIWI	Cell signaling technology	2079	1: 500
mouse anti-Flag	Sigma	F1804	1: 1000
rabbit anti-SYCP3	Novus	NB300-232	1: 500
mouse anti-SYCP3	Santa Cruz	sc-74569	1: 100
goat anti-SYCP3	Santa Cruz	sc-20845	1: 100
mouse anti-γH2AX	Millipore	05-636	1: 500
rabbit anti-γH2AX	Cell signaling technology	20E3	1: 1000
mouse anti-RNA polymerase II	Santa Cruz	sc-47701	1: 100
mouse anti-DMC1	Proteintech	13714-1-AP	1: 500
mouse anti-MLH1	BD Pharmingen	550838	1: 50
rabbit anti-MDC1	Proteintech	24721-1-AP	1: 100
rabbit anti-ATR	Proteintech	19787-1-AP	1: 50
rabbit anti-phospho-Histone H3 (Thr3)(pH3)	Millipore	07-424	1: 1000
mouse anti-α-tubulin	Sigma	T6199	1: 500
rabbit anti-γ-tubulin	Abcam	ab179503	1: 500
human anti-CREST	Immunovision	HCT-0100	1: 500
rabbit anti-CEP192	Proteintech	18832-1-AP	1: 40
Alexa Fluor® 488 conjugated donkey anti-mouse IgG	Invitrogen	A21202	1: 500
Alexa Fluor® 488 conjugated donkey anti-rabbit IgG	Invitrogen	A21206	1: 500
Alexa Fluor® 488 conjugated donkey anti-goat IgG	Invitrogen	A11055	1: 500
FITC–conjugated affinipure goat anti-human IgG	Proteintech	SA00003-12	1: 500
Alexa Fluor® 546 conjugated donkey anti-mouse IgG	Invitrogen	A10036	1: 500
Alexa Fluor® 546 conjugated donkey anti-rabbit IgG	Invitrogen	A10040	1: 500
Alexa Fluor® 594 conjugated goat anti-mouse IgG	Invitrogen	A11001	1: 500
Alexa Fluor® 594 conjugated goat anti-rabbit IgG	Invitrogen	A11012	1: 500
Alexa Fluor® 647 conjugated goat anti-mouse IgG	Invitrogen	A31571	1: 500

**Antibody for WB**			
goat anti-PRRC2A	Santa Cruz	sc-78859	1: 200
mouse anti-Flag	Sigma	F1804	1: 1000
mouse anti-β-actin	Proteintech	60008-1-Ig	1: 5000
rabbit anti-β-actin	ABclonal	AC026	1: 50000
mouse anti-GAPDH	ABclonal	AC033	1: 20000
rabbit anti-CEP152	Proteintech	21815-1-AP	1: 500
mouse anti-CDK1	Abcam	ab18	1: 200
rabbit anti-CCNB1	Cell signaling technology	4138	1: 500
rabbit anti-CCNA2	Abcam	ab181591	1: 2000
rabbit anti-CEP192	Proteintech	18832-1-AP	1: 500
rabbit anti-WNK1	Proteintech	28357-1-AP	1: 10000
goat anti-DAZL	GeneTex	GTX89448	1: 500
rabbit anti-PABPC1	Proteintech	10970-1-AP	1: 1000
rabbit anti-YBX1	Abcam	ab76149	1: 2000
rabbit anti-YBX2	Abcam	ab154829	1: 2000
rabbit anti-FXR1	Proteintech	13194-1-AP	1: 5000
rabbit anti-EIF4G3	Thermo	PA5-31101	1: 5000
rabbit anti-HSPA2	Abcam	ab108416	1: 8000
rabbit anti-DCP1A	Abcam	ab183709	1: 1000
rabbit anti-G3BP1	Proteintech	13057-2-AP	1: 5000
rabbit anti-PATL1	ABclonal	A13170	1: 1000
rabbit anti-DDX4	Abcam	ab13840	1: 1000
rabbit anti-MIWI	Cell signaling technology	2079	1: 1000
HRP-conjugated goat anti-rabbit IgG	Sigma	A6154	1: 5000
HRP-conjugated goat anti-mouse IgG	Sigma	A4416	1: 5000
HRP-conjugated rabbit anti-goat IgG	Sigma	A5420	1: 5000
