# Supplementary material for: The m6A reader PRRC2A is essential for meiosis I completion during spermatogenesis
Source: Nat Commun. 2023 Mar 24;14:1636. doi: 10.1038/s41467-023-37252-y (PMC10039029; doi:10.1038/s41467-023-37252-y)
Supplement: Supplementary file 3 — Description of Additional Supplementary Files [file 41467_2023_37252_MOESM3_ESM.pdf]

Description of Additional Supplementary Files

File Name: Supplementary Data 1
Description: List of RNA-seq & Ribo-seq data

File Name: Supplementary Data 2
Description: PRRC2A RIP-seq

File Name: Supplementary Data 3
Description: PRRC2A RIP-seq&MeRIP-seq

File Name: Supplementary Data 4
Description: Cell-type-specific gene set
