# Supplementary material for: The m6A reader PRRC2A is essential for meiosis I completion during spermatogenesis
Source: Nat Commun. 2023 Mar 24;14:1636. doi: 10.1038/s41467-023-37252-y (PMC10039029; doi:10.1038/s41467-023-37252-y)
Supplement: Supplementary file 5 — Reporting Summary [file 41467_2023_37252_MOESM5_ESM.pdf]

nature portfolio

Corresponding author(s): Fengchao Wang

Last updated by author(s): Feb 25, 2023

Reporting Summary

Nature Portfolio wishes to improve the reproducibility of the work that we publish. This form provides structure for consistency and transparency in reporting. For further information on Nature Portfolio policies, see our Editorial Policies and the Editorial Policy Checklist.

Statistics

For all statistical analyses, confirm that the following items are present in the figure legend, table legend, main text, or Methods section.

n/a	Confirmed	
☐	☒	The exact sample size (*n*) for each experimental group/condition, given as a discrete number and unit of measurement
☐	☒	A statement on whether measurements were taken from distinct samples or whether the same sample was measured repeatedly
☐	☒	The statistical test(s) used AND whether they are one- or two-sided<br>*Only common tests should be described solely by name; describe more complex techniques in the Methods section.*
☒	☐	A description of all covariates tested
☒	☐	A description of any assumptions or corrections, such as tests of normality and adjustment for multiple comparisons
☐	☒	A full description of the statistical parameters including central tendency (e.g. means) or other basic estimates (e.g. regression coefficient) AND variation (e.g. standard deviation) or associated estimates of uncertainty (e.g. confidence intervals)
☐	☒	For null hypothesis testing, the test statistic (e.g. F, t, r) with confidence intervals, effect sizes, degrees of freedom and P value noted<br>*Give P values as exact values whenever suitable.*
☒	☐	For Bayesian analysis, information on the choice of priors and Markov chain Monte Carlo settings
☒	☐	For hierarchical and complex designs, identification of the appropriate level for tests and full reporting of outcomes
☒	☐	Estimates of effect sizes (e.g. Cohen's *d*, Pearson's *r*), indicating how they were calculated

Our web collection on statistics for biologists contains articles on many of the points above.

Software and code

Policy information about availability of computer code

Data collection	OLYMUPUS VS-ASW 2.9 (Olympas), Zen 2.3 (blue edition)(Zeiss), NIS-Elements AR 5.21.00 (Nikon), Bio-Rad CFX Manager 3.1(BioRad), BD FACSDiva software (version 8.0.3) were commercially available software used in data collection.

Data analysis	Microsoft Excel (professional plus 2016)<br>Fiji image J2 software (version1.8.0)<br>Flowjo software (vX.0.7)<br>GraphPad Prism 6 (GraphPad Software)<br>Trim Galore (version 0.6.4)<br>STAR (version 2.7.3a)<br>featureCounts (version 2.0.0)<br>DEseq2 (version 1.30.1)<br>Bowtie2 (version 2.3.5.1)<br>Xtail package (version 1.1.5)<br>MACS2 (version 2.1.2)<br>BEDTools' intersect (version 2.28.0)<br>igvtools (version 2.9.4)<br>HOMER (version 4.11.1)<br>BEDTools' shuffleBed (version 2.28.0)<br>Metascape (http://metascape.org)<br>GSEA software (version 4.1.0)<br>Heatmap plots were plotted on http://www.bioinformatics.com.cn

For manuscripts utilizing custom algorithms or software that are central to the research but not yet described in published literature, software must be made available to editors and reviewers. We strongly encourage code deposition in a community repository (e.g. GitHub). See the Nature Portfolio guidelines for submitting code & software for further information.

Data

Policy information about availability of data

All manuscripts must include a data availability statement. This statement should provide the following information, where applicable:

- Accession codes, unique identifiers, or web links for publicly available datasets
- A description of any restrictions on data availability
- For clinical datasets or third party data, please ensure that the statement adheres to our policy

GRCm38_68 (mm10) and a published dataset (GEO: GSE102346) were used in current study.
All sequencing data generated in this study have been deposited in the Genome Sequence Archive (GSA)(https://ngdc.cncb.ac.cn/gsa/) of China National Center for Bioinformation-National Genomics Data Center (CNCB-NGDC) under accession code: CRA005170. To review all datasets at GSA (CRA005170): Go to https://ngdc.cncb.ac.cn/gsa/s/6WoHg89y. Source data are provided with this paper.

Human research participants

Policy information about studies involving human research participants and Sex and Gender in Research.

Reporting on sex and gender	N/A
Population characteristics	N/A
Recruitment	N/A
Ethics oversight	N/A

Note that full information on the approval of the study protocol must also be provided in the manuscript.

Field-specific reporting

Please select the one below that is the best fit for your research. If you are not sure, read the appropriate sections before making your selection.

☒ Life sciences ☐ Behavioural & social sciences ☐ Ecological, evolutionary & environmental sciences

For a reference copy of the document with all sections, see nature.com/documents/nr-reporting-summary-flat.pdf

Life sciences study design

All studies must disclose on these points even when the disclosure is negative.

Sample size	No statistical method was used to predetermine the sample size. Sample size was determined to reflect biological and technical variance of the investigated parameters based on previously published literature (10.1016/j.jgg.2022.03.005). For all histology, immunofluorescence, western blot, immunoprecipitation, and qPCR experiments, we performed at least three independent biological replicates.
Data exclusions	No data were excluded from analysis.

Replication	All experiments were performed for at least 3 independent biological replicates except for ribo-seq with 2 independent biological replicates, which were specified in figure legends.
Randomization	Samples and cells were randomly allocated into groups. Mice with comparable age and body size were randomly selected and divided into experimental groups.
Blinding	Investigators were blinded to group allocation of experiment and data analysis.

Behavioural & social sciences study design

All studies must disclose on these points even when the disclosure is negative.

Study description	
Research sample	
Sampling strategy	
Data collection	
Timing	
Data exclusions	
Non-participation	
Randomization	

Ecological, evolutionary & environmental sciences study design

All studies must disclose on these points even when the disclosure is negative.

Study description	
Research sample	
Sampling strategy	
Data collection	
Timing and spatial scale	
Data exclusions	
Reproducibility	
Randomization	
Blinding	

Did the study involve field work? ☐ Yes ☐ No

Field work, collection and transport

Field conditions	
Location	
Access & import/export	
Disturbance	

Reporting for specific materials, systems and methods

We require information from authors about some types of materials, experimental systems and methods used in many studies. Here, indicate whether each material, system or method listed is relevant to your study. If you are not sure if a list item applies to your research, read the appropriate section before selecting a response.

Materials & experimental systems

n/a	Involved in the study
☐	☒ Antibodies
☒	☐ Eukaryotic cell lines
☒	☐ Palaeontology and archaeology
☐	☒ Animals and other organisms
☒	☐ Clinical data
☒	☐ Dual use research of concern

Methods

n/a	Involved in the study
☒	☐ ChIP-seq
☐	☒ Flow cytometry
☒	☐ MRI-based neuroimaging

Antibodies

Antibodies used

Antibody for immunohistochemistry
goat anti-PRRC2A Santa Cruz sc-78859 1: 200
rabbit anti-DDX4 Abcam ab13840 1: 1000
rabbit anti-MIWI Cell signaling technology 2079 1: 500
mouse anti-Flag Sigma F1804 1: 1000
rabbit anti-SYCP3 Novus NB300-232 1: 500
mouse anti-SYCP3 Santa Cruz sc-74569 1: 100
goat anti-SYCP3 Santa Cruz sc-20845 1: 100
mouse anti-γH2AX Millipore 05-636 1: 500
rabbit anti-γH2AX Cell signaling technology 20E3 1: 1000
mouse anti-RNA polymerase II Santa Cruz sc-47701 1: 100
mouse anti-DMC1 Proteintech 13714-1-AP 1: 500
mouse anti-MLH1 BD Pharmingen 550838 1: 50
rabbit anti-MDC1 Proteintech 24751-1-AP 1: 100
rabbit anti-ATR Proteintech 19787-1-AP 1: 50
rabbit anti-phospho-Histone H3 (Thr3)(pH3) Millipore 07-424 1: 1000
mouse anti-α-tubulin Sigma T6199 1: 500
rabbit anti-γ-tubulin Abcam ab179503 1: 500
human anti-CREST Immunovision HCT-0100 1: 500
rabbit anti-CEP192 Proteintech 18832-1-AP 1: 40
Alexa Fluor® 488 conjugated donkey anti-mouse IgG Invitrogen A21202 1: 500
Alexa Fluor® 488 conjugated donkey anti-rabbit IgG Invitrogen A21206 1: 500
Alexa Fluor® 488 conjugated donkey anti-goat IgG Invitrogen A11055 1: 500
FITC–conjugated affinipure goat anti-human IgG Proteintech SA00003-12 1: 500
Alexa Fluor® 546 conjugated donkey anti-mouse IgG Invitrogen A10036 1: 500
Alexa Fluor® 546 conjugated donkey anti-rabbit IgG Invitrogen A10040 1: 500
Alexa Fluor® 594 conjugated goat anti-mouse IgG Invitrogen A11001 1: 500
Alexa Fluor® 594 conjugated goat anti-rabbit IgG Invitrogen A11012 1: 500
Alexa Fluor® 647 conjugated goat anti-mouse IgG Invitrogen A31571 1: 500
Antibody for WB
goat anti-PRRC2A Santa Cruz sc-78859 1: 200
mouse anti-Flag Sigma F1804 1: 1000
mouse anti-β-actin Proteintech 60008-1-Ig 1: 5000
rabbit anti-β-actin ABclonal AC026 1: 50000
mouse anti-GAPDH ABclonal AC033 1: 20000
rabbit anti-CEP152 Proteintech 21815-1-AP 1: 500
mouse anti-CDK1 Abcam ab18 1: 200
rabbit anti-CCNB1 Cell signaling technology 4138 1: 500
rabbit anti-CCNA2 Abcam ab181591 1: 2000
rabbit anti-CEP192 Proteintech 18832-1-AP 1: 500
rabbit anti-WNK1 Proteintech 28357-1-AP 1: 10000
goat anti-DAZL GeneTex GTX89448 1: 500
rabbit anti-PABPC1 Proteintech 10970-1-AP 1: 1000
rabbit anti-YBX1 Abcam ab76149 1: 2000
rabbit anti-YBX2 Abcam ab154829 1: 2000
rabbit anti-FXR1 Proteintech 13194-1-AP 1: 5000
rabbit anti-EIF4G3 Thermo PA5-31101 1: 5000
rabbit anti-HSPA2 Abcam ab108416 1: 8000
rabbit anti-DCP1A Abcam ab183709 1: 1000
rabbit anti-G3BP1 Proteintech 13057-21 1: 5000
rabbit anti-PATL1 ABclonal A13170 1: 1000

rabbit anti-DDX4 Abcam ab13840 1: 1000
rabbit anti-MIWI Cell signaling technology 2079 1: 1000
HRP-conjugated goat anti-rabbit IgG Sigma A6154 1: 5000
HRP-conjugated goat anti-mouse IgG Sigma A4416 1: 5000
HRP-conjugated rabbit anti-goat IgG Sigma A5420 1: 5000

Validation

goat anti-PRRC2A Santa Cruz sc-78859 was validated in the previous paper (10.1038/s41422-018-0113-8)
goat anti-SYCP3 Santa Cruz sc-20845 was validated in the previous paper (10.1016/j.jgg.2022.03.005)
Other antibodies were commercially sourced with validation statements available on the following manufactures' websites:
rabbit anti-DDX4 Abcam ab13840 https://www.abcam.com/ddx4--mvh-antibody-ab13840.html
rabbit anti-MIWI Cell signaling technology 2079 https://www.cellsignal.com/products/primary-antibodies/miwi-g82-antibody/2079
mouse anti-Flag Sigma F1804 https://www.sigmaaldrich.com/HK/zh/product/sigma/f1804
rabbit anti-SYCP3 Novus NB300-232 https://www.novusbio.com/products/scp3-sycp3-antibody_nb300-232
mouse anti-SYCP3 Santa Cruz sc-74569 https://www.scbt.com/p/scp-3-antibody-d-1
mouse anti-γH2AX Millipore 05-636 https://www.sigmaaldrich.com/HK/zh/product/mm/05636
rabbit anti-γH2AX Cell signaling technology 20E3 https://www.cellsignal.com/products/primary-antibodies/phospho-histone-h2a-x-ser139-20e3-rabbit-mab/9718
mouse anti-RNA polymerase II Santa Cruz sc-47701 https://www.scbt.com/p/pol-ii-antibody-ctd4h8
mouse anti-DMC1 Proteintech 13714-1-AP https://www.ptglab.com/products/DMC1-Antibody-13714-1-AP.htm
mouse anti-MLH1 BD Pharmingen 550838 https://www.bdbiosciences.com/en-nz/products/reagents/microscopy-imaging-reagents/immunohistochemistry-reagents/purified-mouse-anti-human-mlh-1.550838
rabbit anti-MDC1 Proteintech 24721-1-AP https://www.ptglab.com/products/MDC1-Antibody-24721-1-AP.htm
rabbit anti-ATR Proteintech 19787-1-AP https://www.ptglab.com/products/ATR-Antibody-19787-1-AP.htm
rabbit anti-phospho-Histone H3 (Thr3)(pH3) Millipore 07-424 https://www.sigmaaldrich.com/HK/zh/product/mm/07424
mouse anti-α-tubulin Sigma T6199 https://www.sigmaaldrich.com/FR/fr/product/sigma/t6199
rabbit anti-γ-tubulin Abcam ab179503 https://www.abcam.com/gamma-tubulin-antibody-epr16793-centrosome-marker-ab179503.html
human anti-CREST Immunovision HCT-0100 https://calbiotech.com/products/human-antibody-against-centromere
rabbit anti-CEP192 Proteintech 18832-1-AP https://www.ptgcn.com/products/CEP192-Antibody-18832-1-AP.htm
Alexa Fluor® 488 conjugated donkey anti-mouse IgG Invitrogen A21202 https://www.thermofisher.com/antibody/product/Donkey-anti-Mouse-IgG-H-L-Highly-Cross-Adsorbed-Secondary-Antibody-Polyclonal/A-21202
Alexa Fluor® 488 conjugated donkey anti-rabbit IgG Invitrogen A21206 https://www.thermofisher.com/antibody/product/Donkey-anti-Rabbit-IgG-H-L-Highly-Cross-Adsorbed-Secondary-Antibody-Polyclonal/A-21206
Alexa Fluor® 488 conjugated donkey anti-goat IgG Invitrogen A11055 https://www.thermofisher.com/antibody/product/Donkey-anti-Goat-IgG-H-L-Cross-Adsorbed-Secondary-Antibody-Polyclonal/A-11055
FITC–conjugated affinipure goat anti-human IgG Proteintech SA00003-12 https://www.ptgcn.com/products/Fluorescein-FITC-conjugated-Affinipure-Goat-Anti-Human-IgG-H-L-secondary-antibody.htm
Alexa Fluor® 546 conjugated donkey anti-mouse IgG Invitrogen A10036 https://www.thermofisher.com/antibody/product/Donkey-anti-Mouse-IgG-H-L-Highly-Cross-Adsorbed-Secondary-Antibody-Polyclonal/A10036
Alexa Fluor® 546 conjugated donkey anti-rabbit IgG Invitrogen A10040 https://www.thermofisher.com/antibody/product/Donkey-anti-Rabbit-IgG-H-L-Highly-Cross-Adsorbed-Secondary-Antibody-Polyclonal/A10040
Alexa Fluor® 594 conjugated goat anti-mouse IgG Invitrogen A11001 https://www.thermofisher.com/antibody/product/Goat-anti-Mouse-IgG-H-L-Cross-Adsorbed-Secondary-Antibody-Polyclonal/A-11001
Alexa Fluor® 594 conjugated goat anti-rabbit IgG Invitrogen A11012 https://www.thermofisher.com/antibody/product/Goat-anti-Rabbit-IgG-H-L-Cross-Adsorbed-Secondary-Antibody-Polyclonal/A-11012
Alexa Fluor® 647 conjugated goat anti-mouse IgG Invitrogen A31571 https://www.thermofisher.com/antibody/product/Donkey-anti-Mouse-IgG-H-L-Highly-Cross-Adsorbed-Secondary-Antibody-Polyclonal/A-31571
mouse anti-β-actin Proteintech 60008-1-Ig https://www.ptgcn.com/products/ACTB-Antibody-60008-1-Ig.htm
rabbit anti-β-actin ABclonal AC026 https://abclonal.com/catalog-antibodies/ActinRabbitmAbHighDilution/AC026
mouse anti-GAPDH ABclonal AC033 https://abclonal.com/catalog-antibodies/GAPDHMousemAbHighDilution/AC033
rabbit anti-CEP152 Proteintech 21815-1-AP https://www.ptgcn.com/products/CEP152-Antibody-21815-1-AP.htm
mouse anti-CDK1 Abcam ab18 https://www.abcam.com/cdk1-antibody-a17-ab18.html
rabbit anti-CCNB1 Cell signaling technology 4138 https://www.cellsignal.com/products/primary-antibodies/cyclin-b1-antibody/4138
rabbit anti-CCNA2 Abcam ab181591 https://www.abcam.com/cyclin-a2-antibody-epr17351-ab181591.html
rabbit anti-WNK1 Proteintech 28357-1-AP https://www.ptglab.com/products/WNK1-Antibody-28357-1-AP.htm
goat anti-DAZL GeneTex GTX89448 https://www.genetex.com/Product/Detail/DAZL-antibody-Internal/GTX89448
rabbit anti-PABPC1 Proteintech 10970-1-AP https://www.ptglab.com/products/PABPC1,PABP-Antibody-10970-1-AP.htm
rabbit anti-YBX1 Abcam ab76149 https://www.abcam.com/yb1-antibody-ep2708y-ab76149.html
rabbit anti-YBX2 Abcam ab154829 https://www.abcam.com/msy2ybox2ybx2-antibody-epr28142-ab154829.html
rabbit anti-FXR1 Proteintech 13194-1-AP https://www.ptgcn.com/products/FXR1-Antibody-13194-1-AP.htm
rabbit anti-EIF4G3 Thermo PA5-31101 https://www.thermofisher.com/antibody/product/eIF4G3-Antibody-Polyclonal/PA5-31101
rabbit anti-HSPA2 Abcam ab108416 https://www.abcam.com/hspa2-antibody-epr4596-ab108416.html
rabbit anti-DCP1A Abcam ab183709 https://www.abcam.com/dcp1a-antibody-epr13822-ab183709.html

rabbit anti-G3BP1	Proteintech	13057-2-AP	https://www.ptglab.com/products/G3BP1-Antibody-13057-2-AP.htm
rabbit anti-PATL1	ABclonal	A13170	https://abclonal.com/catalog-antibodies/PATL1PolyclonalAntibody/A13170
HRP-conjugated goat anti-rabbit IgG	Sigma	A6154	https://www.sigmaaldrich.com/US/en/product/sigma/a6154
HRP-conjugated goat anti-mouse IgG	Sigma	A4416	https://www.sigmaaldrich.com/US/en/product/sigma/a4416
HRP-conjugated rabbit anti-goat IgG	Sigma	A5420	https://www.sigmaaldrich.com/US/en/product/sigma/a5420

Eukaryotic cell lines

Policy information about cell lines and Sex and Gender in Research

Cell line source(s)

Authentication

Mycoplasma contamination

Commonly misidentified lines
(See ICLAC register)

Palaeontology and Archaeology

Specimen provenance

Specimen deposition

Dating methods

☐ Tick this box to confirm that the raw and calibrated dates are available in the paper or in Supplementary Information.

Ethics oversight

Note that full information on the approval of the study protocol must also be provided in the manuscript.

Animals and other research organisms

Policy information about studies involving animals; ARRIVE guidelines recommended for reporting animal research, and Sex and Gender in Research

Laboratory animals	All mice in this study were C57BL6 strains. Mice were maintained under specific pathogen-free conditions of a 12 h light/dark cycle at controlled temperature (20-25 °C) and humidity (50-70%) and were provided with food and water ad libitum in the Animal Care Facility at National Institute of Biological Sciences, Beijing. Stra8-Cre mice were C57BL6 strains purchased from Jackson Laboratory and these adult mice were used to mate with Prrc2a fl/fl mice to generate Prrc2a-cko mice.
Wild animals	No wild animals were used in the study.
Reporting on sex	All mice used in the study were male. Our study aimed to examine the regulatory function of PRRC2A in spermatogenesis.
Field-collected samples	No field-collected samples were used in the study.
Ethics oversight	All animal experiments were approved by the Chinese Ministry of Health national guidelines and performed following institutional regulations of Institutional Animal Care and Use Committee at the National Institute of Biological Sciences, Beijing.

Note that full information on the approval of the study protocol must also be provided in the manuscript.

Clinical data

Policy information about clinical studies
All manuscripts should comply with the ICMJE guidelines for publication of clinical research and a completed CONSORT checklist must be included with all submissions.

Clinical trial registration

Study protocol

Data collection

Outcomes

Dual use research of concern

Policy information about dual use research of concern

Hazards

Could the accidental, deliberate or reckless misuse of agents or technologies generated in the work, or the application of information presented in the manuscript, pose a threat to:

No	Yes	
☐	☐	Public health
☐	☐	National security
☐	☐	Crops and/or livestock
☐	☐	Ecosystems
☐	☐	Any other significant area

Experiments of concern

Does the work involve any of these experiments of concern:

No	Yes	
☐	☐	Demonstrate how to render a vaccine ineffective
☐	☐	Confer resistance to therapeutically useful antibiotics or antiviral agents
☐	☐	Enhance the virulence of a pathogen or render a nonpathogen virulent
☐	☐	Increase transmissibility of a pathogen
☐	☐	Alter the host range of a pathogen
☐	☐	Enable evasion of diagnostic/detection modalities
☐	☐	Enable the weaponization of a biological agent or toxin
☐	☐	Any other potentially harmful combination of experiments and agents

ChIP-seq

Data deposition

☐ Confirm that both raw and final processed data have been deposited in a public database such as GEO.

☐ Confirm that you have deposited or provided access to graph files (e.g. BED files) for the called peaks.

Data access links
May remain private before publication.

Files in database submission

Genome browser session
(e.g. UCSC)

Methodology

Replicates

Sequencing depth

Antibodies

Peak calling parameters

Data quality

Software

Flow Cytometry

Plots

Confirm that:

☒ The axis labels state the marker and fluorochrome used (e.g. CD4-FITC).

☒ The axis scales are clearly visible. Include numbers along axes only for bottom left plot of group (a 'group' is an analysis of identical markers).

☒ All plots are contour plots with outliers or pseudocolor plots.

☒ A numerical value for number of cells or percentage (with statistics) is provided.

Methodology

Sample preparation	Adult testes were dissected and tunica albuginea was removed in PBS. Seminiferous tubules of one control testis or two Prrc2a-cko testes were dispersed with tweezers and incubated in 10 ml DMEM medium containing 1 mg/ml Collagenase IV (YEASEN, C3125030) and 0.1 mg/ml DNaseI (YEASEN, D2122070) for 25 min at 34 ℃ with rotation. Tubule fragments were collected by gravity settlement and washed twice with 10 ml PBS. Then, tubules were digested in 10 ml 0.05% Trypsin/EDTA (Gibco, 25300062) containing 0.1 mg/ml DNaseI for 8 min at 34 ℃℃ and were gently pipetted up and down to disperse germ cells. 1ml FBS was added and the cell suspension was passed through nylon mesh with 40 µm pore size (FALCON, 352340). After centrifuging of 300x g for 5 min, germ cells were washed by 1ml DMEM containing 10% FBS and resuspended by 4ml DMEM containing 10% FBS. Then, 4 µl 10 mg/ml Hoechst 33342 (Sigma, B2261) was added, and cells were stained for 60 min at 34 ℃ with rotation. Before sorting, 8 µl 1 mg/ml propidium iodide (Invitrogen, P3566) was added, and the cell suspension was filtered by 40-µm nylon mesh another time. Cell suspensions were sorted by BD FACSAria Fusion-II with the 70 µm nozzle. 355 nm laser was used to excite Hoechst 33342 and fluorescence was recorded with a 450/40 nm band-pass filter (Hoechst blue) and 635 nm long filter (Hoechst red). Spermatocytes were collected in DMEM containing 10% FBS for subsequent experiments.
Instrument	BD FACSAria Fusion-II
Software	BD FACSDiva Software v8.0.3 were used for data collection. Flowjo (vX.0.7) is for data analysis.
Cell population abundance	In adult control testes, L&Z spermatocytes and P&D spermatocytes account for around 8.29% and 7.76% of total cells.<br>In adult Prrc2a-cko testes, L&Z spermatocytes and P&D spermatocytes account for around 20.03% and 18.62% of total cells.<br>In P20 control testes, L&Z spermatocytes and P&D spermatocytes account for around 20.73% and 8.15% of total cells.<br>In P20 Prrc2a-cko testes, L&Z spermatocytes and P&D spermatocytes account for around 21.30% and 4.40% of total cells.<br>The relevant figures and gating strategy were showed in supplementary figure 4d. The detailed gating strategy were reproted in a pervious paper (10.1002/cyto.a.22463).<br>We detected by immunostaining of SYCP3 and γH2A for chromosome spread of sorted spermatocytes and found the purity of each group is more than 90%.
Gating strategy	We adopted the gating strategy in pervious reported method (Gaysinskaya, Valeriya, et al. "Optimized flow cytometry isolation of murine spermatocytes." Cytometry Part A 85.6 (2014): 556-565) with some modification. We grouped leptotene and zygotene spermatocytes into one group named L&Z, and grouped pachytene and diplotene spermatocytes into one group named P&D.

☒ Tick this box to confirm that a figure exemplifying the gating strategy is provided in the Supplementary Information.

Magnetic resonance imaging

Experimental design

Design type	
Design specifications	
Behavioral performance measures	

Acquisition

Imaging type(s)	
Field strength	
Sequence & imaging parameters	
Area of acquisition	

Diffusion MRI ☐ Used ☐ Not used

Preprocessing

Preprocessing software

Normalization

Normalization template

Noise and artifact removal

Volume censoring

Statistical modeling & inference

Model type and settings

Effect(s) tested

Specify type of analysis: ☐ Whole brain ☐ ROI-based ☐ Both

Statistic type for inference
(See Eklund et al. 2016)

Correction

Models & analysis

n/a	Involved in the study
☐	☐ Functional and/or effective connectivity
☐	☐ Graph analysis
☐	☐ Multivariate modeling or predictive analysis

Functional and/or effective connectivity

Graph analysis

Multivariate modeling and predictive analysis
